# Diagnostic performance of next-generation sequencing in thyroid nodules and molecular correlates of tumor aggressiveness in papillary thyroid carcinoma

**DOI:** 10.3389/fendo.2026.1950587

**Published:** 2026-10-14

**Authors:** Songze Zhang, Nan Zhao, Lan Qin, Lin Zheng

**Affiliations:** 1 Department of Thyroid and Breast Surgery, The Affiliated People’s Hospital of Ningbo University, Ningbo, Zhejiang, China; 2 Center for Clinical Genetics and Genomics, Dian Diagnostics Group Co., Ltd., Hangzhou, Zhejiang, China; 3 Department of Clinical Laboratory, The First Affiliated Hospital of Ningbo University, Ningbo, Zhejiang, China

**Keywords:** *BRAF*-like, diagnostic performance, genetic alterations, lymph node metastasis, papillary thyroid carcinoma, *RAS*-like, tumor size

## Abstract

**Background:**

Genetic alterations play a crucial role in the pathogenesis and progression of papillary thyroid carcinoma (PTC). However, the diagnostic and prognostic significance of specific genetic alterations in PTC remains to be elucidated. This study aimed to evaluate the diagnostic performance of next-generation sequencing (NGS)-based genetic testing and to examine associations between genetic alteration subtypes and adverse clinicopathological features in PTC.

**Methods:**

A total of 279 thyroid specimens with NGS-based genetic testing and pathological diagnoses were enrolled, of which 201 specimens with histopathologically confirmed PTC constituted the primary analytical cohort. Diagnostic performance—sensitivity, specificity, positive predictive value (PPV), and negative predictive value (NPV)—for predicting malignancy was assessed using postoperative histopathology as the reference standard, with a positive result defined as detection of a pathogenic or likely pathogenic mutation. Alterations were categorized as *BRAF*-like, *RAS*-like, or Fusion, and their associations with lymph node metastasis (LNM) and tumor size (cutoffs: 0.5 cm, 1 cm) were evaluated using Pearson’s chi-squared test or Fisher’s exact test.

**Results:**

Among the 279 thyroid specimens, 219 had postoperative pathology, of which 203 were malignant, 6 borderline, and 10 benign. *BRAF* V600E was the most frequent genetic alteration (68.10% overall; 79.10% in PTC). Genetic testing demonstrated high sensitivity (86.21%), specificity (90.00%), and PPV (99.43%), but low NPV (24.32%). The Fusion subtype showed a trend toward association with LNM (OR = 7.23, 95% CI 0.85–61.17, *P* = 0.048). *BRAF*-like and *RAS*-like subtypes showed no significant associations with LNM. For tumor size, *BRAF*-like alterations were significantly enriched in tumors > 0.5 cm (OR = 2.60, 95% CI 1.24–5.46, *P* = 0.0098); no significant associations were observed at the 1 cm cutoff. *RAS*-like and Fusion subtypes showed no significant associations with tumor size.

**Conclusion:**

Genetic testing demonstrated high PPV (99.43%) but a low NPV (24.32%), indicating that a positive result identifies malignancy while a negative result cannot exclude cancer. Gene fusions showed a trend toward association with LNM in PTC. *BRAF*-like mutations appeared more often in tumors > 0.5 cm, whereas *RAS*-like alterations bore no clear link to adverse pathological features. These results suggest that genetic profiling may improve diagnosis and risk assessment of PTC.

## 1 Introduction

Papillary thyroid carcinoma (PTC) is the most common endocrine malignancy and accounts for approximately 84% of all thyroid malignancies (1). The rising incidence of PTC has been largely attributed to the increased detection of subclinical, indolent lesions driven by the widespread use of high-resolution ultrasonography and other imaging modalities (2). Although most cases of PTC follow an indolent clinical course and papillary thyroid microcarcinomas (PTMC) (≤ 1 cm) are suitable for active surveillance, adverse pathological features such as lymph node metastasis (LNM) and larger tumor diameter (>1 cm) confer higher risks of locoregional recurrence and poorer outcomes, typically requiring curative surgery. Therefore, accurate preoperative risk stratification for aggressive tumor behavior remains a key unmet clinical need (1).

The molecular landscape of PTC is now well characterized. *BRAF* V600E represents the most frequent driver alteration in PTC, reported in 61.7% of cases in Western cohorts (3) and up to 70–80% in Asian populations, with 72.4% observed in a Chinese cohort (4), and a summary frequency of 68.7% across Asian studies, reaching as high as 87.1% in individual Korean cohorts (5). This is followed by *RAS* mutations (~10%) and gene fusions involving *RET* or *NTRK* (~5–10%) in Western cohorts; these drivers are generally regarded as mutually exclusive (3). The Cancer Genome Atlas (TCGA) classified PTC into two transcriptome-based subtypes: *BRAF*-like (BL) and *RAS*-like (RL), each showing distinct biological behaviors and gene expression profiles (6). Despite this genetic clarity, the clinical utility for risk prediction in PTC remains unsettled. A recent meta-analysis of 20,570 PTC patients found that the *BRAF* V600E mutation was associated with higher risk of LNM and recurrence, but not with distant metastasis or mortality, underscoring its limited independent prognostic value (7). Another cohort study of 393 PTC patients (tumors ≤ 15 mm) showed no connection between the *BRAF* V600E mutation and high-risk clinicopathological features, even though the mutation was strongly associated with larger tumor size (OR = 4.92, 95% CI 2.40–10.07, *P* < 0.001) (8). The prognostic significance of gene fusions has been less thoroughly examined. In a study of 150 young adult PTC patients (aged 17–35 years), fusion oncogenes involving *RET*, *NTRK3*, or *BRAF* were significantly associated with lateral LNM, with risk escalating in younger age groups (75.00% for patients < 25 years vs. 27.27% for 25–29 years vs. 8.33% for 30–35 years, *P* = 0.037) (9).

Next-generation sequencing (NGS) genetic testing has demonstrated clinical utility as a diagnostic tool for thyroid nodules (10, 11), with heritable genetic factors also contributing to thyroid cancer and benign nodular disease risk (12). A single-center study evaluating NGS testing in thyroid nodules found that the combination of NGS and cytology could facilitate accurate diagnosis, particularly for indeterminate nodules (13). A subsequent study further demonstrated that integrating genetic alterations with radiomic features significantly improved the prediction of LNM (AUC increased from 71.5% to 87.0%) (14). However, further study is needed to evaluate how these NGS panels perform in real-world clinical cohorts, particularly how reliably a negative result can rule out cancer.

To address these gaps, we conducted a retrospective cohort study of 279 patients who underwent NGS-based genetic testing of thyroid specimens, with a primary analysis of 201 patients with histopathologically confirmed PTC. Our study had two principal aims (1) to evaluate the diagnostic accuracy of NGS-based genetic testing for distinguishing malignant from benign thyroid lesions, using postoperative histopathology as the reference standard; and (2) to investigate the associations of distinct molecular subtypes—*BRAF*-like, *RAS*-like, and Fusion—with adverse pathological features, specifically LNM and maximum tumor diameter (tumor size). Through these analyses, we sought to clarify the clinical value of molecular subtyping for both diagnostic refinement and risk stratification in PTC.

## 2 Materials and methods

### 2.1 Patients

A total of 279 patients who underwent genetic testing of thyroid specimens between June 2023 and March 2025 at The First Affiliated Hospital of Ningbo University were retrospectively identified; all patients had complete genetic alteration profiles generated by NGS. Available pathological diagnostic records were retrieved for all patients and categorized according to the highest level of pathological confirmation obtained (postoperative histopathology > intraoperative frozen section > cytopathology). Among the 279 patients, 219 had definitive postoperative histopathological diagnoses, 33 had only intraoperative frozen section diagnoses, and 27 had only cytopathological diagnoses from fine-needle aspiration.

For subsequent analyses, the cohort was restricted to the 219 patients with postoperative histopathological confirmation because only definitive histopathology provides a reliable diagnostic reference standard and complete staging data. Within this group, 203 patients were diagnosed with malignant lesions, 10 with benign lesions, and 6 with borderline lesions. Of the 203 malignant cases, 201 cases were histopathologically confirmed as PTC and constituted the analytical cohort. The remaining 2 malignant cases, which corresponded to other histological subtypes, were excluded from further analysis.

Clinicopathological data, including LNM status and maximum tumor diameter, were extracted from surgical pathology reports for the 201 PTC patients. The patient enrollment and subgroup stratification workflow is illustrated in **Figure 1**.

**Figure 1:**
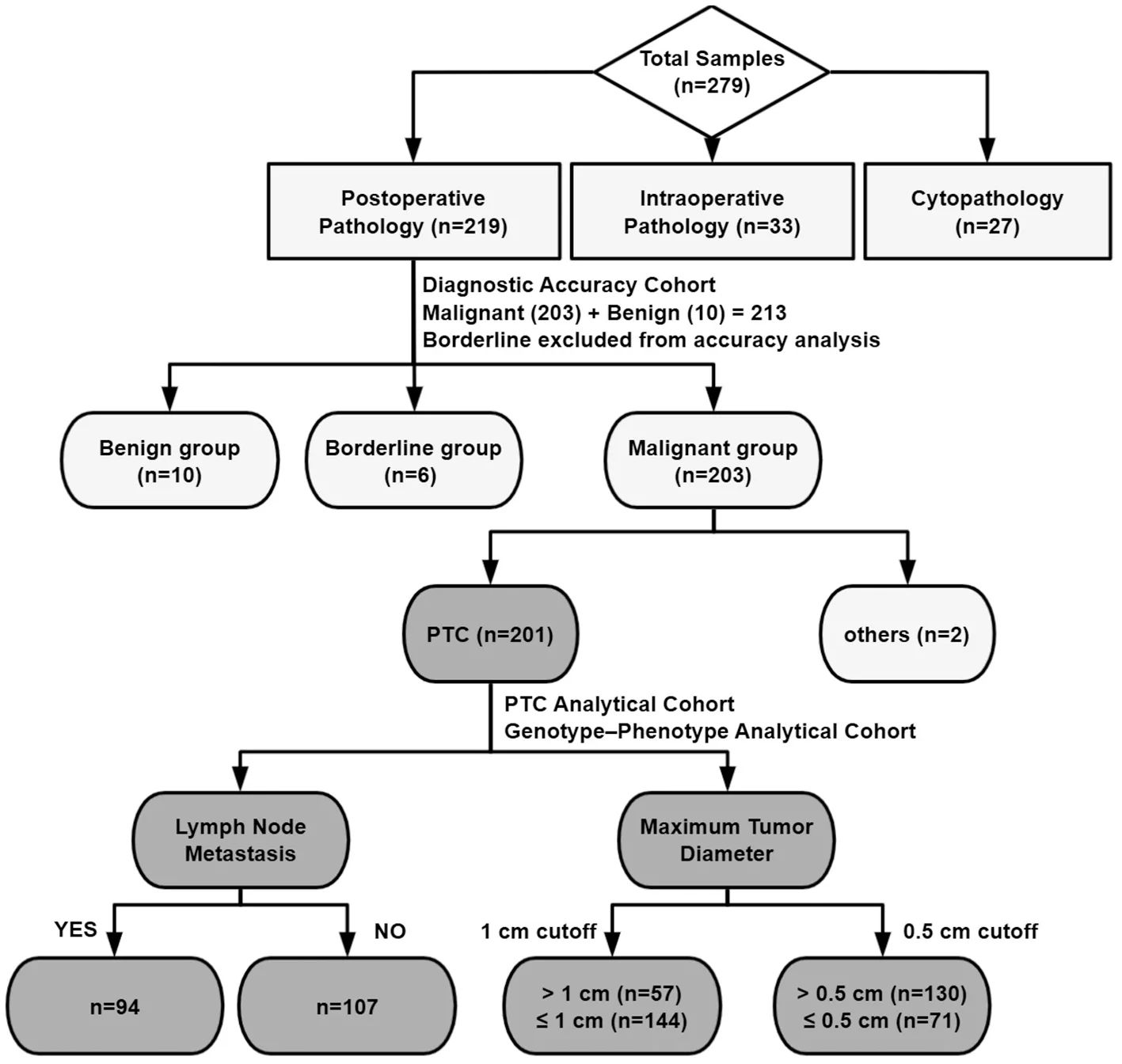
Flow diagram of patient enrollment and subgroup stratification.

### 2.2 Next-generation sequencing

Genomic profiling was performed on DNA extracted from patient specimens using a targeted NGS panel for thyroid cancer (Dian Diagnostics, Hangzhou, China) on the NovaSeq 6000 System (Illumina, Inc., San Diego, CA). The panel interrogated a total of 11 genes relevant to thyroid carcinogenesis. Specifically, full exonic sequencing was applied to 10 genes (*BRAF*, *HRAS*, *KRAS*, *NRAS*, *NTRK1*, *NTRK3*, *PAX8*, *RET*, *THADA*, and *TP53*) for the detection of single nucleotide variants (SNVs) and small insertions/deletions (INDELs). In addition, the promoter region of *TERT* was sequenced. The panel also enabled the detection of fusions involving five genes (*NTRK1*, *NTRK3*, *PAX8*, *RET*, and *THADA*). The procedure included DNA extraction, fragmentation, end repair, adapter ligation, and target enrichment via hybrid capture.

### 2.3 Bioinformatics analysis

Tumor samples achieved a mean coverage depth of approximately 1000×. Mutations were called at a variant allele frequency (VAF) threshold of 1%. Sequencing data were converted to FASTQ format and aligned to the human reference genome hg19 (GRCh37) using the Burrows-Wheeler Aligner (BWA, version 0.7.17) software. For the detection of SNVs and INDELs, MuTect2 (version 4.2) and Pisces (version 5.3.0.0) were employed. For the identification of gene fusions, STAR-Fusion (version 1.12.0) and Arriba (version 2.1.0) were utilized. Candidate fusion transcripts were validated by manual review and visualization using Integrative Genomics Viewer (IGV version 2.16.2).

Due to the lack of germline sequencing data, a rigorous filtering strategy was applied to exclude potential germline variants. Variants with a minor allele frequency (MAF) ≥ 1% in population databases (ESP, 1000 Genomes Project, ExAC, gnomAD) or those classified as “benign” or “likely benign” in ClinVar were removed. The remaining variants were classified according to the Association for Molecular Pathology (AMP), American Society of Clinical Oncology (ASCO), and College of American Pathologists (CAP) guidelines.

### 2.4 Statistical analysis

Diagnostic performance of genetic testing for differentiating malignant from benign thyroid lesions was evaluated using definitive postoperative histopathology as the reference standard. Sensitivity, specificity, positive predictive value (PPV), and negative predictive value (NPV) were calculated. For all diagnostic accuracy measures, 95% confidence intervals (CIs) were estimated using the Wilson score method.

For genotype-phenotype association analyses, the cohort was restricted to the 201 patients with histopathologically confirmed PTC. Each genetic alteration subtype (*BRAF*-like, *RAS*-like, and Fusion) was analyzed independently as a binary variable (presence vs. absence of the alteration). Associations between genetic subtypes and categorical clinicopathological outcomes were assessed using the two-sided Pearson’s chi-squared test; when the expected frequency in any cell of a contingency table was less than 5, the two-sided Fisher’s exact test was used instead. Odds ratios (ORs) with corresponding 95% CIs were reported to quantify the strength of associations. ORs and 95% CIs were calculated using the Woolf (logit) method based on asymptotic normality.

All statistical analyses were performed using SPSS version 26.0 (IBM Corp., Armonk, NY, USA). Statistical significance was set at a two-tailed *P* < 0.05.

### 2.5 Molecular subtype definitions

Molecular subtypes were defined according to the framework established by The Cancer Genome Atlas (TCGA) for PTC and adopted by the WHO Classification of Thyroid Neoplasms, adapted to the gene coverage of the targeted NGS panel used in this study. In the WHO classification, the *BRAF*-like subtype encompasses *BRAF* V600E and gene fusions involving *ALK*, *BRAF*, *RET*, *NTRK1/3*, and *MET*. The *RAS*-like subtype encompasses *RAS* mutations (*NRAS*, *KRAS*, *HRAS*), *BRAF* non-V600E mutations (e.g., K601E), alterations in *DICER1*, *EZH1*, *EIF1AX*, *PTEN*, and gene fusions in *PPARG* and *THADA*. *BRAF* non-V600E mutations are classified as *RAS*-like because they signal through CRAF-dependent activation of the MAPK pathway, a mechanism analogous to that of *RAS* mutations (3, 15).

Because the targeted NGS panel used in this study interrogated a defined set of 11 genes, the operational definitions were necessarily restricted to the genes covered by the panel. Operational definitions used in this study:

*BRAF*-like subtype: cases harboring *BRAF* V600E, *RET* fusions, *NTRK1* fusions, or *NTRK3* fusions.

*RAS*-like subtype: cases harboring *NRAS*, *KRAS*, or *HRAS* mutations, *BRAF* non-V600E mutations, *PAX8* fusions, or *THADA* fusions.

Fusion subtype: cases harboring *RET*, *NTRK1*, *NTRK3*, *PAX8*, or *THADA* fusions (analyzed separately from *BRAF*-like and *RAS*-like). This group was defined to examine fusion-specific associations with clinicopathological features, as fusion-driven PTC may have distinct metastatic behavior.

## 3 Results

### 3.1 Clinicopathological characteristics and baseline demographics of the study cohort

A total of 279 thyroid specimens were included in the study cohort. The distribution of diagnostic specimen types was as follows: postoperative pathology in 219 patients (78.49%), intraoperative pathology in 33 patients (11.83%), and cytopathology in 27 patients (9.68%). The cohort included 193 females (69.18%) and 86 males (30.82%). Age at diagnosis showed a broad distribution, with the highest proportion in the 50–59 years age group (73/279, 26.16%), followed by 30–39 years (70/279, 25.09%), 40–49 years (50/279, 17.92%), 60–69 years (40/279, 14.34%), < 30 years (32/279, 11.47%), and ≥ 70 years (14/279, 5.02%).

Based on the final pathological assessment, among the 279 specimens, 240 (86.02%) were assigned to the malignant group, 14 (5.02%) to the borderline group, and 25 (8.96%) to the benign group. The malignant group accounted for 203 of 219 (92.69%) in postoperative pathology, 31 of 33 (93.94%) in intraoperative pathology, and 6 of 27 (22.22%) in cytopathology. The borderline group comprised 6 of 219 (2.74%) in postoperative pathology, 1 of 33 (3.03%) in intraoperative pathology, and 7 of 27 (25.93%) in cytopathology. The benign group included 10 of 219 (4.57%) in postoperative pathology, 1 of 33 (3.03%) in intraoperative pathology, and 14 of 27 (51.85%) in cytopathology.

Regarding genetic alterations, the most frequent mutation was *BRAF* V600E, detected in 190 of 279 patients (68.10%, 186 isolated, 3 co-mutated with *TERT*, and 1 co-mutated with *KRAS*) of the total cohort, with the highest prevalence in the postoperative pathology group (159/219, 72.60%, 156 isolated, 2 co-mutated with *TERT*, and 1 co-mutated with *KRAS*), followed by the intraoperative pathology group (21/33, 63.64%, 20 isolated, 1 co-mutated with *TERT*) and the lowest in the cytopathology group (10/27, 37.04%). Co-mutations included *BRAF* V600E combined with *TERT* (3/279, 1.08%), *BRAF* V600E with *KRAS* (1/279, 0.36%), *TERT* with *NRAS* (1/279, 0.36%), and *TERT* with *TP53* (1/279, 0.36%). *BRAF* non-V600E mutations were present in 2 patients (0.72%). *RET* fusions were identified in 8 patients (2.87%), and *NTRK1* fusions in 2 patients (0.72%). Single *RAS* mutations included *NRAS* (9/279, 3.23%) and *KRAS* (4/279, 1.43%). Variants of uncertain significance (VUS) were observed in 6 patients (2.15%). Notably, 56 patients (20.07%) had no detectable genetic alterations (negative results), with the highest negative rate in cytopathology (15/27, 55.56%) compared to postoperative pathology (37/219, 16.89%) and intraoperative pathology (4/33, 12.12%). Detailed clinicopathological and genetic characteristics of all patients, stratified by diagnostic group, are summarized in **Table 1**.

**Table 1:** Clinicopathological characteristics and genetic alterations of 279 patients subdivided into postoperative pathology, intraoperative pathology, and cytopathology groups.

Characteristic	Total (n=279)	Postoperative pathology (n=219, 78.49%)	Intraoperative pathology (n=33, 11.83%)	Cytopathology (n=27, 9.68%)
Gender				
Male	86 (30.82%)	65 (29.68%)	9 (27.27%)	12 (44.44%)
Female	193 (69.18%)	154 (70.32%)	24 (72.73%)	15 (55.56%)
Age at diagnosis (years)				
<30	32 (11.47%)	22 (10.05%)	3 (9.09%)	7 (25.93%)
30-39	70 (25.09%)	51 (23.29%)	12 (36.36%)	7 (25.93%)
40-49	50 (17.92%)	39 (17.81%)	6 (18.18%)	5 (18.52%)
50-59	73 (26.16%)	63 (28.77%)	7 (21.21%)	3 (11.11%)
60-69	40 (14.34%)	33 (15.07%)	4 (12.12%)	3 (11.11%)
≥ 70	14 (5.02%)	11 (5.02%)	1 (3.03%)	2 (7.41%)
Surgical pathology				
Malignant group	240 (86.02%)	203 (92.69%)	31 (93.94%)	6 (22.22%)
Borderline group	14 (5.02%)	6 (2.74%)	1 (3.03%)	7 (25.93%)
Benign group	25 (8.96%)	10 (4.57%)	1 (3.03%)	14 (51.85%)
Genetic alterations				
*BRAF* V600E^a^	186 (66.67%)	156 (71.23%)	20 (60.61%)	10 (37.04%)
*BRAF* V600E*+TERT*	3 (1.08%)	2 (0.91%)	1 (3.03%)	0 (0%)
*BRAF* V600E*+KRAS*	1 (0.36%)	1 (0.46%)	0 (0%)	0 (0%)
*BRAF* non-V600E	2 (0.72%)	2 (0.91%)	0 (0%)	0 (0%)
*TERT+NRAS*	1 (0.36%)	1 (0.46%)	0 (0%)	0 (0%)
*TERT+TP53*	1 (0.36%)	0 (0%)	1 (3.03%)	0 (0%)
*RET* fusion	8 (2.87%)	6 (2.74%)	2 (6.06%)	0 (0%)
*NTRK1* fusion	2 (0.72%)	1 (0.46%)	1 (3.03%)	0 (0%)
*NRAS*	9 (3.23%)	6 (2.74%)	2 (6.06%)	1 (3.70%)
*KRAS*	4 (1.43%)	2 (0.91%)	2 (6.06%)	0 (0%)
Variant of uncertain significance	6 (2.15%)	5 (2.28%)	0 (0%)	1 (3.70%)
Negative	56 (20.07%)	37 (16.89%)	4 (12.12%)	15 (55.56%)

^a^ The total frequency of *BRAF* V600E mutation (including co-mutations) was 68.10% (190/279) in the total cohort, 72.60% (159/219) in the postoperative pathology group and 63.64% (21/33) in the intraoperative pathology group.

### 3.2 Diagnostic performance of genetic testing in the postoperative pathology cohort

Diagnostic accuracy of genetic testing for differentiating malignant from benign thyroid lesions was evaluated using postoperative histopathology as the reference standard. Among the 219 patients with definitive postoperative pathology, 203 were diagnosed as malignant, 6 as borderline, and 10 as benign; the borderline group was excluded from the diagnostic accuracy analysis. For this analysis, a positive NGS result was defined as the detection of a pathogenic or likely pathogenic mutation, whereas cases with only VUS or no detectable alterations were classified as negative. The sensitivity of genetic testing for identifying malignancy was 86.21% (175/203; 95% CI 80.79%–90.28%), and the specificity was 90.00% (9/10; 95% CI 59.58%–98.21%). The PPV was 99.43% (175/176; 95% CI 96.85%–99.90%), indicating that a positive genetic result is highly predictive of malignancy. In contrast, the NPV was 24.32% (9/37; 95% CI 13.36%–40.12%), reflecting that a negative genetic result does not reliably exclude malignancy. The wide CI for specificity was attributable to the small number of benign cases (n = 10). The single benign case with a pathogenic mutation was diagnosed as nodular goiter with fibrosis and focal follicular epithelial hyperplasia on postoperative histopathology and harbored an NRAS c.181C>A (p.Q61K) mutation. Thus, a positive genetic result was strongly associated with malignancy, whereas a negative result did not reliably rule out malignancy. The detailed data are presented in **Table 2**.

**Table 2:** Diagnostic performance of genetic testing.

Category	With pathogenic mutation	Without pathogenic mutation	Total	
Postoperative Pathology Malignant	175	28	203	Sensitivity with 95% CI 86.21% (175/203; 80.79%–90.28%) Specificity with 95% CI 90.00% (9/10; 59.58%–98.21%) PPV with 95% CI 99.43% (175/176; 96.85%–99.90%) NPV with 95% CI 24.32% (9/37; 13.36%–40.12%)
Postoperative Pathology Benign	1	9	10	
Total	176	37	213	

Borderline cases (n = 6) were excluded, leaving 213 patients (203 malignant, 10 benign) for analysis.

### 3.3 Landscape of genetic alterations in the PTC cohort

Among the 219 patients in the postoperative pathology group, 201 were pathologically confirmed with PTC. Of these, 175 of 201 patients (87.06%) harbored at least one genetic mutation, while 26 (12.94%) had no detectable mutations. The most frequent genetic alteration was *BRAF* V600E, detected in 159 patients (79.10%, 156 isolated, 2 co-mutated with *TERT*, and 1 co-mutated with *KRAS*). Co-occurring alterations included *BRAF* V600E with *TERT* in 2 patients (1.00%; V600E+C228T, n = 1; V600E+C250T, n = 1) and *BRAF* V600E with *KRAS* Q61H in 1 patient (0.50%). One non-V600E *BRAF* mutation (L485_T488delinsF) was identified (0.50%). Gene fusions were observed in 7 patients: *RET* fusions in 6 patients (2.99%), comprising *CCDC6-RET* (C1:R12, n = 2; C8:R12, n = 1), *NCOA4-RET* (N8:R12, n = 2), and *ERC1-RET* (E7:R12, n = 1); and an *IRF2BP2-NTRK1* fusion (I2:N9) in 1 patient (0.50%). *NRAS* mutations were detected in 4 patients (1.99%; Q61R, n = 3; Q61K, n = 1) and *KRAS* mutations in 2 patients (1.00%; G12A, n = 1; Q61K, n = 1). VUS were present in 2 patients (1.00%). The detailed genetic mutation spectrum of the PTC cohort is presented in **Table 3**.

**Table 3:** Genetic alterations of PTC patients in the postoperative pathology group (n=201).

Genetic mutations	PTC patients (n=201)	Mutation/fusion
*BRAF* V600E	156 (77.61%)^a^	V600E (n=156)
*BRAF* V600E*+TERT*	2 (1.00%)	V600E + C228T (n=1) V600E + C250T (n=1)
*BRAF* V600E*+KRAS*	1 (0.50%)	V600E + Q61H (n=1)
*BRAF* non-V600E	1 (0.50%)	L485_T488delinsF (n=1)
*RET* fusion	6 (2.99%)	*CCDC6-RET* (C1:R12) (n=2) *CCDC6-RET* (C8:R12) (n=1) *NCOA4-RET* (N8:R12) (n=2) *ERC1-RET* (E7:R12) (n=1)
*NTRK1* fusion	1 (0.50%)	*IRF2BP2-NTRK1* (I2:N9) (n=1)
*NRAS*	4 (1.99%)	Q61R (n=3) Q61K (n=1)
*KRAS*	2 (1.00%)	G12A (n=1) Q61K (n=1)
Variant of uncertain significance	2 (1.00%)	*RET* S462L (n=1) *NTRK1* A30S (n=1)
Negative	26 (12.94%)	–

^a^ The total frequency of *BRAF* V600E mutation (including co-mutations) in the PTC cohort was 79.10% (159/201).

### 3.4 Association of genetic alteration profiles with LNM: distinct roles of the *BRAF*-like subtype, *RAS*-like subtype, and fusion subtype

We next examined whether specific genetic alteration subtypes were individually associated with the presence of LNM in our cohort of 201 PTC patients. Molecular subtypes were operationally defined as described in Methods (Section 2.5). Each subtype was analyzed independently as a binary variable (presence vs. absence of the alteration).

As summarized in **Table 4**, the Fusion subtype was more frequently observed in patients with LNM (6.38%, 6/94) than in those without LNM (0.93%, 1/107). This finding showed a trend toward statistical significance (OR = 7.23, 95% CI 0.85–61.17, *P* = 0.048). However, the wide CI, driven by the small number of fusion-positive cases, warrants cautious interpretation.

**Table 4:** Association of genetic alteration subtypes with LNM in 201 PTC patients.

Genetic alterations	Metastasis (n=94)	Non-metastasis (n=107)	OR (95% CI) ^*^	*P*-value ^†^
*BRAF*-like	81 (86.17%)	85 (79.44%)	1.61 (0.76–3.42)	0.21
*RAS*-like	1 (1.06%)	6 (5.61%)	0.18 (0.02–1.53)	0.12
Fusion ^‡^	6 (6.38%)	1 (0.93%)	7.23 (0.85–61.17)	**0.048**

*OR is presented with the non-metastasis group as the reference.

^†^*P*-values are from two-sided Pearson’s chi-squared test or two-sided Fisher’s exact test when expected cell frequency <5; significant values (<0.05) are in bold.

^‡^The Fusion subtype comprised all fusions detected (*RET*, n=6; *NTRK1*, n=1). Subtypes were analyzed independently and are not mutually exclusive.

In contrast, neither the *BRAF*-like subtype nor the *RAS*-like subtype showed a statistically significant association with metastatic status. The *BRAF*-like subtype was detected in 86.17% (81/94) of patients with LNM and 79.44% (85/107) of those without LNM; no statistically significant association was observed (OR = 1.61, 95% CI 0.76–3.42, *P* = 0.21). For the *RAS*-like subtype, the prevalence was notably lower in the LNM group (1.06%, 1/94) than in the non-LNM group (5.61%, 6/107), but this difference did not reach statistical significance (OR = 0.18, 95% CI 0.02–1.53, *P* = 0.12).

### 3.5 Correlation between genetic alterations and tumor size: *BRAF*-like mutations predominant in tumors >0.5 cm

To evaluate the association between specific genetic alteration subtypes (*BRAF*-like, *RAS*-like, and Fusion) and tumor size, we analyzed a cohort of 201 PTC patients using two clinically relevant cutoffs for tumor size: 1 cm and 0.5 cm. The genetic subtypes were defined as described in Section 3.4. Each subtype was analyzed independently as a binary variable (presence vs. absence of the alteration) using two-sided Fisher’s exact test. In both analyses, the smaller tumor size group (≤1 cm or ≤0.5 cm) served as the reference category.

#### 3.5.1 Analysis using a 1 cm cutoff

When dichotomized at 1 cm, the cohort comprised 144 patients with tumors ≤1 cm and 57 patients with tumors >1 cm. As shown in **Table 5**, none of the genetic subtypes exhibited a statistically significant association with tumor size at this cutoff. The *BRAF*-like subtype was detected in 85.96% (49/57) of tumors >1 cm and 81.25% (117/144) of tumors ≤1 cm (OR = 1.41, 95% CI 0.60–3.33, *P* = 0.43). The *RAS*-like subtype was present in 5.26% (3/57) of tumors >1 cm and 2.78% (4/144) of tumors ≤1 cm (OR = 1.94, 95% CI 0.42–9.02, *P* = 0.41). The Fusion subtype tended to be more frequent in tumors >1 cm (7.02%, 4/57) than in those ≤1 cm (2.08%, 3/144), but this difference did not reach statistical significance (OR = 3.55, 95% CI 0.77–16.38, *P* = 0.10).

**Table 5:** Association of genetic alteration subtypes with tumor size (1 cm cutoff) in 201 PTC patients.

Genetic alteration	> 1 cm (n=57)	≤ 1 cm (n=144)	OR (95% CI) ^*^	*P*-value ^†^
*BRAF*-like	49 (85.96%)	117 (81.25%)	1.41 (0.60–3.33)	0.43
*RAS*-like	3 (5.26%)	4 (2.78%)	1.94 (0.42–9.02)	0.41
Fusion ^‡^	4 (7.02%)	3 (2.08%)	3.55 (0.77–16.38)	0.10

*OR is presented with the smaller tumor size group as the reference.

^†^*P*-values are from two-sided Pearson’s chi-squared test or two-sided Fisher’s exact test when expected cell frequency <5; significant values (<0.05) are in bold.

^‡^The Fusion subtype comprised all fusions detected (*RET*, n=6; *NTRK1*, n=1). Subtypes were analyzed independently and are not mutually exclusive.

#### 3.5.2 Analysis using a 0.5 cm cutoff

We further refined the analysis using a 0.5 cm cutoff (≤0.5 cm, n = 71; >0.5 cm, n = 130). At this cutoff, a significant association emerged for the *BRAF*-like subtype. As summarized in **Table 6**, *BRAF*-like alterations were significantly enriched in tumors >0.5 cm compared to those ≤0.5 cm (87.69% vs. 73.24%; OR = 2.60, 95% CI 1.24–5.46, *P* = 0.0098). In contrast, the *RAS*-like subtype showed no significant association with tumor size (3.08% vs. 4.23%; OR = 0.72, 95% CI 0.16–3.31, *P* = 0.704). The Fusion subtype tended to be more frequent in tumors >0.5 cm (4.62% vs. 1.41%), though this difference did not reach statistical significance (OR = 3.39, 95% CI 0.40–28.70, *P* = 0.243).

**Table 6:** Association of genetic alteration subtypes with tumor size (0.5 cm cutoff) in 201 PTC patients.

Genetic alteration	> 0.5 cm (n=130)	≤ 0.5 cm (n=71)	OR (95% CI) ^*^	*P*-value ^†^
*BRAF*-like	114 (87.69%)	52 (73.24%)	2.60 (1.24–5.46)	**0.0098**
*RAS*-like	4 (3.08%)	3 (4.23%)	0.72 (0.16–3.31)	0.704
Fusion ^‡^	6 (4.62%)	1 (1.41%)	3.39 (0.40–28.70)	0.243

*OR is presented with the smaller tumor size group as the reference.

^†^*P*-values are from two-sided Pearson’s chi-squared test or two-sided Fisher’s exact test when expected cell frequency <5; significant values (<0.05) are in bold.

^‡^The Fusion subtype comprised all fusions detected (*RET*, n=6; *NTRK1*, n=1). Subtypes were analyzed independently and are not mutually exclusive.

## 4 Discussion

In this retrospective cohort study of 279 patients who received NGS-based genetic testing for thyroid lesions, we evaluated the diagnostic performance of NGS in differentiating malignant from benign thyroid lesions and further explored the associations between molecular subtypes and adverse pathological features in 201 PTC patients. Our principal findings were threefold (1): NGS-based testing demonstrated high PPV (99.43%) but limited NPV (24.32%) for diagnosing malignancy (2); gene fusions in PTC patients showed a trend toward association with an increased risk of LNM (OR = 7.23, *P* = 0.048) (3); *BRAF*-like mutations were significantly enriched in tumors >0.5 cm (OR = 2.60, *P* = 0.0098), whereas *RAS*-like alterations showed no significant association with either LNM or tumor size.

The diagnostic performance observed in our cohort is consistent with previous studies assessing NGS panels for thyroid nodules. The high PPV of 99.43% aligns with previous studies, including a multicenter study of 827 thyroid nodules in which a targeted four-gene NGS panel achieved a PPV of 99.62% for cytologically indeterminate nodules (Bethesda III–V) (16) and a single-center evaluation demonstrating a PPV of 95.0% using a multigene NGS assay in indeterminate thyroid nodules (13). These data collectively support the clinical utility of a positive NGS result as a reliable predictor of malignancy. Of note, this high PPV was obtained in a cohort where 92.69% of cases with definitive postoperative pathology were malignant. The high PPV therefore partly reflects the malignancy-enriched composition of this cohort rather than the intrinsic discriminatory capacity of the NGS panel alone. However, the low NPV (24.32%) in our cohort underscores a critical limitation: a negative genetic result does not reliably exclude thyroid cancer and therefore cannot be used as a standalone basis to defer or avoid surgery. This is consistent with the finding that the NPV of NGS panels may not be sufficiently high to serve as a standalone ‘rule-out’ test to safely obviate diagnostic surgery (17). This limitation is largely attributable to the fact that a subset of PTC lack identifiable driver mutations, as reflected by the 12.94% driver-negative rate in our PTC cohort. For instance, a large cohort study of 855 PTC patients found that 5.26% (45/855) had no detectable genetic mutations by NGS (18). Although the driver-negative rate in our cohort was higher (12.94% vs. 5.26%), this discrepancy may be attributable to differences in panel coverage and the inclusion criteria of the respective study populations. The inclusion of additional oncogenic drivers, such as *EIF1AX*, *AKT1*, and *PIK3CA*, in future panel designs may improve sensitivity. Nevertheless, these findings caution against relying solely on a negative NGS result to defer surgery in cytologically suspicious nodules. Despite the limited NPV, a positive NGS result may still be clinically useful in supporting decisions to proceed with surgery, particularly for indeterminate nodules where additional diagnostic certainty is most needed.

The spectrum of genetic alterations observed in our PTC cohort is broadly consistent with the established molecular epidemiology of PTC, as reflected in recent updates to the classification of thyroid neoplasms (19). *BRAF* V600E was the dominant alteration (79.10%), a prevalence comparable to the 72.4% reported in a large Chinese cohort (4) and the 68.7% pooled prevalence in Asian populations (5). The 2.99% prevalence of *RET* fusions in our cohort is comparable to the 2.8% reported in a similarly sized Chinese PTC cohort (8). The frequency of *RAS* mutations (2.99%) is also consistent with studies from East Asian populations (4, 5). Both frequencies are lower than those reported in Western populations (3). Notably, the 12.94% driver-negative rate in our PTC cohort is consistent with the approximately 13% of PTC that lack known driver mutations as characterized in large-scale genomic studies (20), highlighting the need for continued expansion of sequencing panels to capture rare or novel oncogenic events.

We observed a trend toward an association between the Fusion subtype and LNM (OR = 7.23, 95% CI 0.85–61.17, *P* = 0.048). The result suggests a potentially elevated risk, consistent with emerging evidence that fusion-driven PTC are more prone to lymphatic dissemination. However, the limited number of fusion-positive cases warrants cautious interpretation. A study of pediatric PTC patients reported that fusion oncogenes, particularly those involving *RET* and *NTRK*, are associated with a higher proportion of large tumors (>2 cm), extrathyroidal extension, and lymph node and lung metastases (21). Similarly, *RET* fusions have been identified as the second most frequent alteration (17%) after *BRAF* in a cohort of 160 PTC patients with lateral neck LNM, and patients harboring *RET* fusions presented with more metastatic lymph nodes than those with *BRAF* mutations (22). The biological basis for the heightened metastatic propensity of fusion-positive PTC may involve sustained and potent activation of the MAPK pathway, as *RET* and *NTRK* fusions result in ligand-independent dimerization and constitutive kinase activity (20). *NTRK* fusion-positive thyroid carcinomas have been shown to be significantly associated with LNM, providing further evidence for the aggressive behavior of fusion-driven tumors (23). Of particular relevance to our findings, a large real-world molecular profiling study (n=1,348) observed that *RAS* family mutations and *RET*/*BRAF*/*ETV6* gene fusions were predominantly detected in *BRAF* wild-type PTC, suggesting a molecularly distinct subset of tumors that is largely non-overlapping with *BRAF*-driven disease (24). This observation underscores the importance of comprehensive NGS testing—covering both point mutations and gene fusions—to accurately classify the molecular subtype of each tumor and identify patients who may benefit from more extensive initial surgery.

Regarding *BRAF*-like alterations, we did not observe a significant association between *BRAF*-like alterations and LNM (OR = 1.61, *P* = 0.21). This finding adds to the ongoing controversy surrounding the prognostic value of *BRAF* V600E in PTC. An earlier meta-analysis of 15 studies including 4,909 PTC patients reported that the *BRAF* V600E mutation was significantly associated with LNM (OR = 1.34; 95% CI 1.09–1.65, *P* = 0.005), and the association was even stronger in the PTMC subgroup (OR = 3.49; 95% CI 2.02–6.02, *P* < 0.00001) (25). The most recent and comprehensive meta-analysis encompassing 46 studies and 20,570 patients confirmed that *BRAF* V600E mutation is significantly associated with an increased risk of nodal metastasis and showed a borderline association with recurrence; however, it was not significantly associated with distant metastasis or mortality (7). These findings reinforce the notion that *BRAF* V600E alone is insufficient to independently predict long-term clinical outcomes. Further evidence for this controversy comes from the real-world analysis of 1,348 patients, in which *BRAF* V600E mutation status was not significantly associated with overall survival, despite its enrichment in PTC (68.4%) and its association with a pro-inflammatory tumor microenvironment (24). Several factors may explain the discrepancy across studies. First, the inclusion of fusion-positive cases within our *BRAF*-like group may have confounded the analysis, as fusions are associated with higher metastatic risk. Second, the prognostic impact of *BRAF* V600E may be context-dependent, modified by factors such as tumor size, histological variant, and the presence of cooperating genetic events—particularly *TERT* promoter mutations. Notably, despite the debated prognostic value of *BRAF* V600E, its therapeutic relevance is supported by preclinical models demonstrating sensitivity of *BRAF* V600E-mutant thyroid cancer spheroids to combined *BRAF* and MEK inhibition (26).

The synergistic effect of *BRAF* V600E and *TERT* promoter co-mutations on the aggressiveness of PTC has been well documented in the literature. In a retrospective cohort of 507 PTC patients, those with coexisting *BRAF V600E* and *TERT* C228T mutations exhibited the most aggressive phenotype, with a recurrence rate of 68.6%, compared with only 8.7% in patients harboring neither mutation (HR = 8.51, 95% CI 4.84–14.97) (27). A meta-analysis of 13 studies including 4,347 PTC patients further demonstrated that concurrent *BRAF* V600E and *TERT* promoter mutations were associated with higher risks of recurrence (HR = 6.60, 95% CI 3.82–11.40) and PTC-related mortality (HR = 20.07, 95% CI 8.37–48.09) than either mutation alone (28). In a more recent cohort of PTC patients, *BRAF* V600E and *TERT* C228T co-mutations were identified in 10.96% of cases, and co-mutated patients exhibited older onset age, higher MACIS scores, larger tumor size, greater radioiodine resistance, and significantly poorer overall and progression-free survival compared with those harboring single or no mutations (29). Consistently, a study of recurrent PTC demonstrated that the coexistence of *BRAF* V600E and *TERT* promoter mutations independently predicted disease progression after reoperation, with a hazard ratio of 5.44 (95% CI 2.21–13.3) (30). In our cohort, only 2 of 201 PTC patients (1.00%) harbored concurrent *BRAF* V600E and *TERT* promoter mutations, which precluded a meaningful subgroup analysis of this double-mutant population. Given the well-established synergistic effect of the co-mutations, the very low frequency of *BRAF*/*TERT* co-mutations in our cohort may partially explain why BRAF-like alterations were not significantly associated with LNM or other high-risk features. Future studies enriched for *BRAF*/*TERT* co-mutated cases are needed to fully characterize the prognostic significance of these cooperative genomic events.

The analysis of tumor size yielded an exploratory finding: *BRAF*-like mutations were significantly enriched in tumors >0.5 cm, yet no significant association was observed at the 1 cm cutoff. This suggests that *BRAF*-like mutations may play a role in early tumor growth; however, this interpretation should be viewed with caution. A previous study of 393 PTC patients with tumors ≤15 mm found that the *BRAF* V600E mutation was correlated with larger tumor diameter (OR = 4.92, 95% CI 2.40–10.07, *P* < 0.001) yet was not significantly associated with high-risk clinicopathological features. Furthermore, tumor size ≥5 mm was the independent predictor of high-risk PTMC (OR = 2.55, 95% CI 1.57–4.14, *P* < 0.001) (8). It should be noted that Jin et al. examined *BRAF* V600E specifically, whereas the present study assessed a broader *BRAF*-like category. A recent study developed a nomogram for predicting central LNM in which age, tumor size, and *BRAF* V600E status were independent predictors (AUC = 0.710), suggesting that *BRAF* V600E may have prognostic value when integrated with clinicopathological parameters rather than as a standalone biomarker (31). The absence of a significant association at the 1 cm threshold in our study may reflect the smaller number of tumors >1 cm (n=57) in our cohort. A retrospective study comparing two active surveillance criteria suggested that tumor size >0.5 cm was a significant risk factor for central lymph node metastasis in low-risk PTMC patients (32), supporting the potential clinical relevance of the 0.5 cm threshold.

The *RAS*-like subtype did not demonstrate significant associations with either LNM or tumor size in our cohort. This is consistent with the characterization of *RAS*-like PTC as generally indolent tumors with a low propensity for nodal metastasis. However, the small number of *RAS*-like cases in our cohort (n=7) limits definitive conclusions. Furthermore, a recent study revealed that while *RAS*-like PTC typically follow a more indolent clinical course, a subset can progress through immune evasion mechanisms, which are distinct from those in *BRAF*-like tumors (6). Larger studies enriched for *RAS*-mutant PTC are needed to fully characterize the clinical behavior of this subtype.

Several limitations of this study should be acknowledged. First, the retrospective, single-center design may introduce selection bias into our findings. Second, the small number of cases in the *RAS*-like (n=7) and Fusion (n=7) subgroups limited statistical power, reflected in the wide CI for the corresponding OR estimates. The association between Fusion and LNM, although consistent with the literature, should be interpreted with caution given the small sample size and should be regarded as hypothesis-generating only. Third, the targeted NGS panel used in this study covered 11 genes; rare oncogenic drivers such as *EIF1AX*, *AKT1*, and *PIK3CA* were not interrogated, which may have contributed to the 12.94% driver-negative rate and the low NPV observed in our cohort. Fourth, the exclusion of cases without definitive postoperative histopathology reduced the benign group in the diagnostic accuracy analysis to only 10 cases, resulting in a wide CI for specificity (59.58%–98.21%). Therefore, the specificity estimate should not be used in isolation as a measure of diagnostic performance. Fifth, long-term follow-up data, including disease-free survival and recurrence, were not available for the majority of patients, precluding analysis of the prognostic impact of molecular subtypes on clinical outcomes. Finally, the low prevalence of *TERT* promoter mutations in our cohort (1.00%) prevented a meaningful analysis of the synergistic effect of *BRAF*/*TERT* co-mutations, which has been shown to be a critical determinant of aggressive disease in recent studies (29, 30).

## 5 Conclusion

In this retrospective cohort study of 279 patients undergoing NGS-based genetic testing for thyroid lesions, the diagnostic accuracy analysis (n=213) demonstrated high PPV (99.43%) but limited NPV (24.32%) for identifying malignancy, indicating that a positive result may support the decision to proceed with surgery, whereas a negative result cannot be used as a standalone basis to defer or avoid surgery. In the PTC cohort (n=201), gene fusions showed a trend toward association with LNM (OR = 7.23, 95% CI 0.85–61.17, *P* = 0.048); however, this finding is limited by the small number of fusion-positive cases (n=7) and should be regarded as hypothesis-generating only. *BRAF*-like alterations were significantly enriched in tumors > 0.5 cm (OR = 2.60, 95% CI 1.24–5.46, P = 0.0098), whereas no significant association was observed at the 1 cm threshold; *RAS*-like alterations showed no significant associations with either LNM or tumor size. These findings suggest that comprehensive molecular profiling may complement cytological and histopathological assessment in the diagnostic evaluation of thyroid nodules, but larger prospective studies with long-term follow-up are needed to validate the prognostic significance of molecular subtypes in PTC.

## Data Availability

The datasets presented in this study can be found in online repositories. The names of the repository/repositories and accession number(s) can be found below: https://www.ncbi.nlm.nih.gov/, PRJNA1520010.
